# Who uses self-management apps from the OncoAppstore: description of characteristics, symptoms, and functioning of a real-world user population

**DOI:** 10.1007/s11764-025-01855-9

**Published:** 2025-07-05

**Authors:** A. M. de Korte, L. van Deursen, M. L. van der Lee, J. J. Aardoom, N. P. M. Ezendam, P. Heine, F. Mols, H. Mulder-Mertens, M. M. Stouten, C. R. M. Lammens

**Affiliations:** 1 The Netherlands Comprehensive Cancer Organisation, Utrecht, The Netherlands; 2 CoRPS - Centre of Research on Psychological Disorders and Somatic Diseases, Department of Medical and Clinical Psychology, Tilburg University, Tilburg, The Netherlands; 3 Department of National Health and Care, Center for Public Health, Care and Society, National Institute for Public Health and the Environment, Bilthoven, The Netherlands; 4 National eHealth Living Lab, Leiden, The Netherlands; 5 Scientific Research Department, Centre for Psycho-Oncology, Helen Dowling Institute, Bilthoven, The Netherlands; 6 Department of Public Health and Primary Care, Leiden University Medical Center, Leiden, The Netherlands; 7 Stichting kanker.nl, Amsterdam, The Netherlands; 8 Dutch Cancer Society, Amsterdam, The Netherlands; 9 National AYA “Young and Cancer” Network, Amsterdam, The Netherlands

**Keywords:** EHealth, MHealth, Oncology, Self-management, Quality of life, Quality of care

## Abstract

**Purpose:**

The rising prevalence of cancer in the Netherlands challenges resource allocation and sustainable care delivery. eHealth interventions, such as self-management applications, may reduce healthcare utilisation. To enhance access to self-management tools, the OncoAppstore was launched in July 2023. This study evaluates the sociodemographic and health-related quality of life (HRQoL) characteristics of OncoAppstore users.

**Methods:**

User data from the OncoAppstore and HRQoL data were integrated with clinical data from the Netherlands Cancer Registry. Descriptive analyses were used to describe sociodemographic and clinical characteristics. Chi-square and Mann-Whitney *U* tests compared OncoAppstore users with the Dutch adult cancer population.

**Results:**

A total of 3620 users requested a health credit, and 4333 applications were purchased. Most users were highly educated (61%), had breast cancer (50%) and a high socioeconomic position (53%). Compared to non-users, OncoAppstore users were younger (56 vs 69), predominantly female (78% vs 55%), more often received primary treatment, and were diagnosed earlier. The majority indicated clinically significant distress levels and approximately half reported substantial functional impairments. Symptom burden was highest in the fatigue, pain, and nausea/vomiting domains.

**Conclusion and Implications for Cancer Survivors:**

The OncoAppstore is primarily utilised by highly educated users who seek applications targeting cancer-related fatigue. Insights into the characteristics of users who are receptive to online self-management tools contribute to the improvement of self-management infrastructure and its long-term integration into Dutch cancer care. By providing self-management tools to those who are able to use it, we can potentially save scarce resources for those who are unable to profit from self-management alone.

## Background

The prevalence of cancer in the Netherlands continues to rise, driven by increasing incidence rates, advancements in screening technologies, an ageing population, and more effective treatment options that extend survival [1]. While these developments represent significant progress in oncology, they also contribute to an increasing burden on the healthcare system [2]. The growing population of people directly affected by cancer often faces physical and psychological consequences related to cancer and its treatments [3, 4]. This results in elevated healthcare needs compared to the general population, contributing to challenges in resource allocation and sustainable delivery of care [5–7].

To address these challenges, eHealth interventions, such as digital self-management applications, have emerged as a promising solution. These interventions can facilitate self-management and personalised care, potentially reducing regular healthcare utilisation and alleviating issues such as cancer-related fatigue, pain, depressive symptoms, and improving lifestyle and knowledge [8–18]. Regardless of their demonstrated effectiveness in improving a range of health outcomes, the adoption of digital self-management interventions remains limited. This may be attributed to barriers such as restricted access and availability of some of the self-management applications due to fragmented application supply, poor findability, and the need for a subscription and/or referral by a healthcare professional. Additionally, several challenges among people directly affected by cancer contribute to this underutilisation as well, including limited awareness, perceptions of inferiority compared to in-person care, and reluctance to invest in digital tools [19].

To improve access to self-management applications for Dutch people directly affected by cancer and their relatives, the Dutch Cancer Society (KWF), Stichting kanker.nl (cancer.nl foundation), and the Netherlands Comprehensive Cancer Organisation (IKNL) have launched the “OncoAppstore” within the Dutch online platform “Kanker.nl” in July 2023. Reliable self-management applications were made available and could be purchased with a one-time health credit provided to any interested Dutch person directly affected by cancer and their relatives. This study aims to provide information on the use of the OncoAppstore and evaluate sociodemographic factors and health-related quality of life (HRQoL) variables of OncoAppstore users directly affected by cancer. Insights into the use of the OncoAppstore and its user population could be relevant to improve self-management application infrastructure and sustainability in cancer care.

## Methods

### Design

This prospective observational real-world study is part of the OncoAppstore study. A detailed description of the methods of the OncoAppstore study can be found in the protocol paper [20]. The study was designed to compare characteristics and HRQoL of OncoAppstore users with non-buyers. Non-buyers were defined as those who requested a health credit but did not buy an application. However, the vast majority of OncoAppstore participants purchased at least one application (91%); therefore, the non-buyer group was too small to compare to the user group. Consequently, this study compared characteristics of people directly affected by cancer who completed the baseline (T0) questionnaire (users) to the general Dutch cancer population (non-users). The study was submitted and exempt from review by the Medical Research Ethics Committee Brabant (NW2022-42), as this study is not subject to the Dutch Medical Research Involving Human Subjects Act. The study adheres to the Declaration of Helsinki [21].

### Setting

The OncoAppstore was developed to improve access to cancer-specific and cancer-relevant self-management applications for people directly affected by cancer and their relatives. The OncoAppstore was launched within the Dutch online platform “Kanker.nl,” as the platform is widely known among people directly affected by cancer, their relatives, and healthcare professionals. The platform has over 1,000,000 visits per month and more than 39,500 individuals with an account. The OncoAppstore is further promoted through information on websites of hospitals, cancer patient organisations, and application suppliers, (social) media campaigns, and through presentations for healthcare professionals. All applications included in the OncoAppstore were tested by project team members (AK and MS) and the Dutch Regional Public Health Service (GGD) on GGD assessment criteria, which include usability, reliability, privacy, and scientific foundation [22].

Users were offered an online health credit of 100 euros, valid for 6 months, to encourage the use of applications, with no personal costs involved. A referral from a healthcare professional was not necessary for participation; thus, anyone could use the OncoAppstore. To access the applications, users had to create an account on “kanker.nl,” request the health credit, and select applications that suited their needs. User data from all participants utilising the health credit was collected, with personal information excluded to maintain anonymity. Additional data were gathered through the Patient Reported Outcomes Following Initial Treatment and Long-term Evaluation of Survivorship (PROFILES) registry [14], a system designed to manage cancer patient data collected via web-based questionnaires and integrate it with clinical data.

### Population and recruitment

The study population consists of OncoAppstore users who requested the health credit, purchased at least one application, and filled in the baseline questionnaire between July 2023 and October 2024. Since only 2% were relatives, we focus on users directly affected by cancer. While requesting the health credit, users were asked if they could be approached for research for this study. If they agreed, users received a patient information letter and informed consent form via the PROFILES registry via email. By signing the consent form, users agreed that their data can be linked to Statistic Netherlands and the Netherlands Cancer Registry (NCR). A detailed description of the use of Statistic Netherlands data for the OncoAppstore study can be found in the paper by van Deursen et al. [23]. After obtaining the informed consent, users were invited by email to fill in a web-based questionnaire (baseline) directly after purchasing the first application. In the event of a non-response, a reminder email was sent a week after the invitation email. Non-users were all people directly affected by cancer, diagnosed between 1989, the first year the NCR collected nationwide data of Dutch cancer patients, and October 2024. Non-users were excluded if they were deceased prior to the OncoAppstore launch, were younger than 18 years old, did not meet the NCR inclusion criteria [24], did not have a malignant tumour, or when their NCR registration was incomplete.

### Outcome measures

*User data* was collected from all OncoAppstore users. The data includes general information on the total number of users and which and how often applications were purchased. Additionally, user-specific data was gathered pertaining to which users requested a health credit in the last week, whether the user purchased at least one application, which applications were purchased per user, and the amount of remaining health credit after 6 months.

Data collected via the PROFILES registry questionnaire at baseline included self-reported sociodemographic (e.g., gender, age, educational level, marital status) and clinical characteristics (e.g., cancer diagnosis (yes/no), tumour recurrence (yes/no), co-morbidities). Moreover, the questionnaire consisted of the distress thermometer (DT) and the problem list [25], questions on employment status, and the European Organisation for Research and Treatment of Cancer Quality of Life Questionnaire Core (EORTC QLQ-C30) [26]. Subsequently, the computerised adaptive test (CAT) version of the EORTC Core was used instead of the 30-item version after baseline data was gathered to guide the design of the EORTC CAT Core [26–28].

The *DT* is a single-item measure of distress experienced in the last week, ranging from 0 (no distress) to 10 (extreme distress) [25] and the clinical cut-off score is 4 [25]. The problem list measured dichotomous responses (yes/no) on 47 items and focused on practical problems (7 items), family/social problems (3 items), emotional problems (9 items), religious/spiritual problems (2 items), and physical problems (25 items). Additionally, a question about wanting to talk to a healthcare professional was included.

Information on *employment status* was collected using questions about current occupation and occupation prior to cancer diagnosis.

Domain-specific *T*-scores for the *EORTC CAT Core* were calculated using EORTC conversion tables. The scales derived from the EORTC CAT item banks are scored using *T*-scores based on item response theory, standardised so that the European general population has a mean of 50 and a standard deviation of 10 for all domains [27]. High scores on functional domains indicate good functioning, whereas high scores on symptom domains reflect high symptom burden. Thresholds for clinical importance for each domain, developed by Giesinger et al., were used to identify clinically significant functional health impairments and symptoms [29].

Clinical data (e.g., tumour type, date of cancer diagnosis, histological classification, clinical stage, primary treatment, socioeconomic position) from the *NCR* of the Netherlands Comprehensive Cancer Organisation was used to link to user data. The NCR routinely collects data on all newly diagnosed cancer patients in the Netherlands since 1989.

### Statistical analysis

Statistical analyses of the data were performed using Statistical Analyses Software version 9.4 (SAS Institute, Cary NC, USA). A descriptive analysis was used to describe the sociodemographic data and clinical characteristics. These variables were compared at baseline between the OncoAppstore users and general Dutch cancer population (non-users) using chi-square analyses for categorical variables and Mann-Whitney *U* tests for continuous variables. A two-sided *p*-value of 5% was considered significant. Means (M), medians (Md), standard deviations (SD), and interquartile ranges (IQR) were used to describe the EORTC CAT Core scores and DT scores at baseline for the OncoAppstore users. The normality of all scores was assessed using the Kolmogorov–Smirnov and Shapiro–Wilk tests.

## Results

### OncoAppstore use

**Fig. 1:**
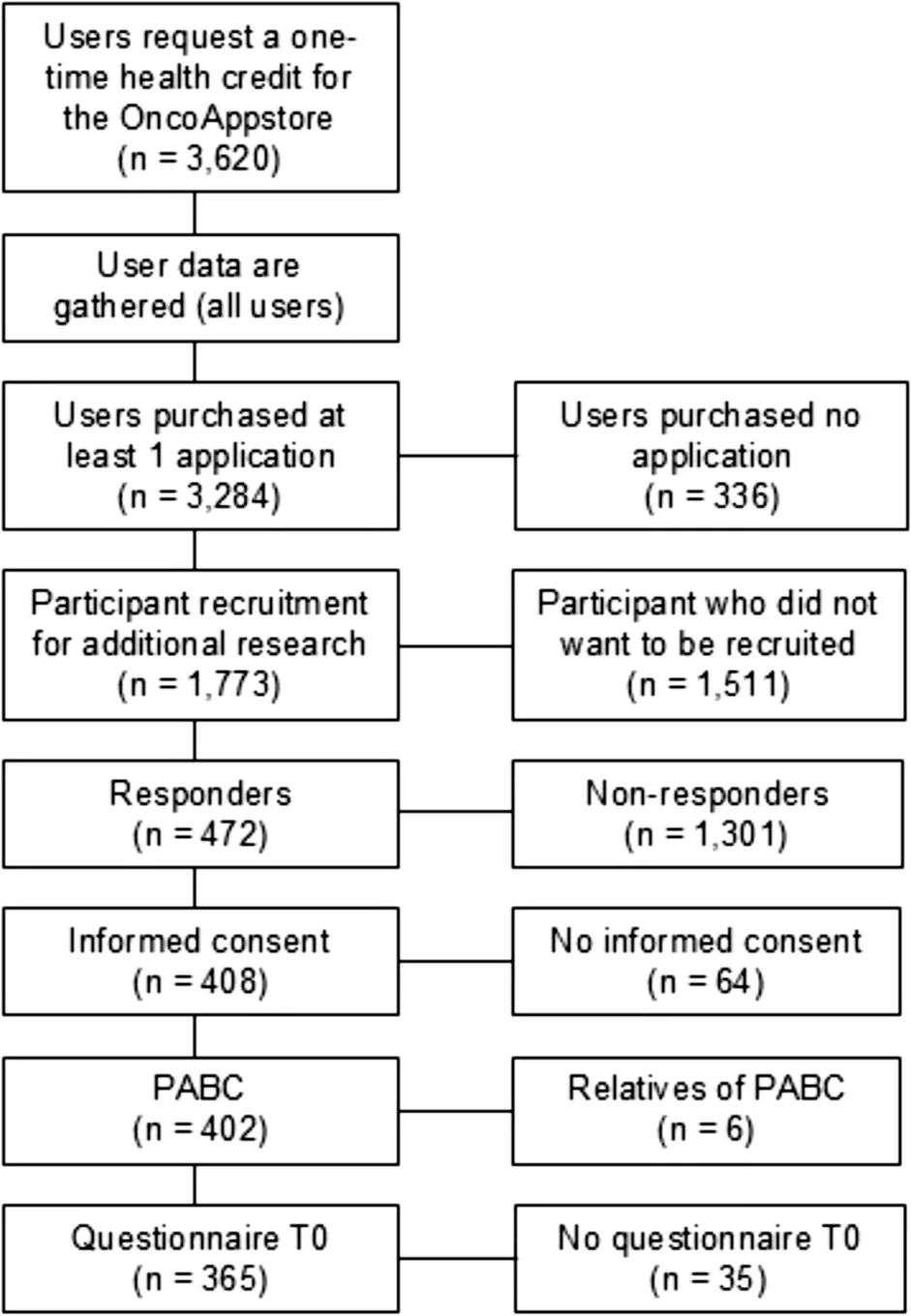
Flowchart. PABC, people directly affected by cancer; T0, baseline

The OncoAppstore went live in July 2023. Between then and October 2024, 3620 users requested a health credit for the OncoAppstore. With the health credit, 3284 users (91%) purchased at least one application and from that group, 1773 users agreed to be approached for additional questionnaires for this study (54%). 365 users directly affected by cancer provided informed consent and filled in the baseline questionnaire (10% of all participants who requested a health credit). Figure 1 shows the flowchart of this study.

In total, 4333 applications were purchased by 3284 users between the launch of the OncoAppstore and October 2024. This is on average 1.3 applications per user. The 10 most purchased applications are listed in Table 1. The most purchased application, Untire Now, focuses on cancer-related fatigue [8]. Moreover, in October 2024, the health credit of 1739 users had expired. On average, these users had spent 53.10 euros of their health credit.

**Table 1:** Most purchased applications between the launch of OncoAppstore in July 2023 and October 2024

Purchased applications	Number of purchases
Untire Now [8]	2222
Cancer aftercare guide [17]	197
Help after treatment [48]	164
Self-compassion [48]	154
Breast cancer decision aid—additional treatment [49]	127
Breath [16]	123
Breast cancer decision aid—surgery [49]	111
Healthy nutrition [50]	100
Lifestyle [48]	96
Physical activity [50]	70

### Study population

About half of the 365 users who had filled in the baseline questionnaire were diagnosed with breast cancer, received chemotherapy and/or radiotherapy as primary treatment, and had a high socioeconomic position (Table 2). Moreover, the majority of users were highly educated, had undergone surgery, had no cancer recurrence, and had a partner. The proportion of users who learned about the OncoAppstore via healthcare professionals, friends and family, or the (social) media campaigns continuously expanded and exceeded the proportion of users who found the OncoAppstore via kanker.nl. Of the 365 users, 77% reported having used one or more applications they had purchased.

**Table 2:** Baseline characteristics of participants and non-users

**Characteristics**	**OncoAppstore users *n* (%) (*n* = 365)**	**Non-users *n* (%) (*n* = 966,753)**	***p*-value**
**Age** (mean (SD) median [IQR])^a^	55.9 (10.4)	69.0 (13.5)	**<0.01**^b^
	57.0 [50.0–63.0]	71.0 [61.0–78.0]	
<40	23 (6.3)	33,990 (3.5)	
40–54	127 (34.8)	102,252 (10.6)	
55–69	183 (50.1)	302,638 (31.3)	
70–84	32 (8.8)	430,725 (44.6)	
≥85	0 (0.0)	97,148 (10.1)	
**Age at diagnosis** (mean (SD) median (IQR])	53.6 (10.4)	60.0 (14.9)	**<0.01**^b^
	54.0 [47.0–61.0]	62.0 [51.0–71.0]	
**Gender**			**<0.01**^c^
Female	284 (77.8)	529,576 (54.8)	
**Socioeconomic position**^d^			**<0.01**^**c**^
Low	57 (15.9)	190,919 (26.3)	
Middle	112 (31.3)	260,931 (36.0)	
High	189 (52.8)	273,503 (37.7)	
**Tumour site**^e^			**<0.01**^**c**^
Breast	182 (49.9)	225,550 (23.3)	
Digestive system	41 (11.2)	152,627 (15.8)	
Haematology	39 (10.7)	96,275 (10.0)	
Male genitalia	25 (6.9)	155,869 (16.1)	
Female genitalia	22 (6.0)	54,234 (5.6)	
Respiratory system	15 (4.1)	49,349 (5.1)	
Urinary tract	15 (4.1)	43,985 (4.6)	
Skin	10 (2.7)	123,843 (12.8)	
Head and neck	5 (1.4)	24,835 (2.6)	
Sarcoma	4 (1.1)	15,741 (1.6)	
Endocrine glands	3 (0.8)	12,904 (1.3)	
Central nervous system	2 (0.6)	6759 (0.7)	
Eye	0 (0.0)	2878 (0.3)	
Other	2 (0.6)	1904 (0.2)	
**Tumour stage**^f^			**<0.01**^**c**^
0	2 (0.6)	76 (<0.1)	
I	85 (23.3)	397,078 (41.1)	
II	102 (27.9)	207,718 (21.5)	
III	61 (16.7)	116,779 (12.1)	
IV	36 (9.9)	62,900 (6.5)	
Unknown	13 (3.6)	8890 (0.9)	
Not applicable	66 (18.1)	173,312 (17.9)	
**Time since diagnosis** (years)^a^ median [IQR]	1.0 [0.0–2.0]	6.0 [2.0–13.0]	**<0.01**^b^
<1	156 (42.7)	141,501 (14.6)	
1–2	86 (23.6)	73,592 (7.6)	
2–3	40 (11.0)	64,304 (6.7)	
3–4	20 (5.5)	54,377 (5.6)	
4–5	15 (4.1)	54,357 (5.6)	
>5	48 (13.2)	578,622 (59.9)	
**Primary treatment**^g^			
Surgery	255 (70.1)	528,435 (54.7)	**<0.01**^**c**^
Chemotherapy	189 (51.9)	241,621 (25.0)	**<0.01**^**c**^
Radiotherapy	183 (50.3)	320,319 (33.1)	**<0.01**^**c**^
Hormone therapy	124 (34.1)	152,183 (15.7)	**<0.01**^**c**^
Targeted therapy	72 (19.8)	71,879 (7.4)	**<0.01**^**c**^
Other	21 (5.8)	52,436 (5.4)	0.77^c^
Unknown	0 (0.0)	995 (0.1)	0.54^c^
**Partner status**^hi^			n.a.
Partner	304 (84.4)		
**Level of education**^hj^			n.a.
Low	40 (11.1)		
Middle	100 (27.8)		
High	220 (61.1)		
**Cancer recurrence**^h^			n.a.
Yes	63 (17.5)		
**Co-morbidities**^h^			n.a.
None	157 (43.6)		
1	95 (26.4)		
≥ 2	108 (30.0)		
**How did you find the OncoAppstore?**^h^			n.a.
Via kanker.nl	170 (47.1)		
Via healthcare professionals/hospital	106 (29.4)		
Via friends/family	25 (6.9)		
Via occupational health and safety service	2 (0.6)		
Via general practitioner	1 (0.2)		
Via employer	1 (0.2)		
Other	56 (15.5)		

OncoAppstore users: OncoAppstore users who completed baseline questionnaire (T0)

Non-users: Netherlands Cancer Registry population

^a^At baseline (T0) for participants; at launch of the OncoAppstore (01-07-2023) for non-users

^b^Mann-Whitney *U* tests

^c^Chi^2^ test

^d^Based on median household income in their postal code area

^e^Patients with multiple primary tumours were grouped by their latest diagnosed tumour before OncoAppstore use (participants) or OncoAppstore launch (non-users)

^f^Tumour stage at diagnosis. Ann Arbor staging system and FIGO staging system not included, those malignancies were classified as “not applicable”

^g^Multiple treatment options possible per patient; therefore, percentages do not add up to 100%

^h^No data for non-users available

^i^Partner: married or living together; no partner: divorced, widower, single

^j^Level of education was classified as low, middle, and high according to Statistics Netherlands’ classification [51]

Missing values participants: socioeconomic position (*n* = 7), primary treatment (*n* = 1), partner status (*n* = 5), level of education (*n* = 5), cancer recurrence (*n* = 4), co-morbidities (*n* = 5), how did you find the OncoAppstore? (*n* = 4)

Missing values non-users: socioeconomic position (*n* = 241,400), primary treatment (*n* = 42)

Note: the percentages do not always add to 100% due to rounding

Abbreviations: *SD* standard deviation, *IQR* interquartile range, *n.a.* not applicable

There were statistically significant differences (<0.01) between the users and non-users. The users were on average younger (56 vs 69), more often female (77.8% vs 54.8%), had a different distribution of tumour type, socioeconomic position, and tumour stage, where people with a high socioeconomic position form the largest proportion of users (52.8% vs 37.7%). They were diagnosed more recently and at a younger age compared to non-users. Additionally, there was a statistically significant association between primary treatment and OncoAppstore usage. Users received significantly more surgery, chemotherapy, radiotherapy, hormone therapy, and targeted therapy than non-users.

### Distress thermometer

The median user score (Md = 6.0, IQR [4.0–7.0]) is above the clinical cut-off score (Table 3). The majority of users (72.1%) score above the clinical cut-off, indicating a high level of distress in the study population. Additionally, more than half of users reported experiencing problems with work/school/study (51.3%), memory (57.8%), self-confidence (50.0%), tension/nervousness (58.4%), concentration (70.5%), sleep (61.0%), fatigue (84.7%), out of shape/condition (76.0%), and muscle strength (65.9%).

**Table 3:** Distress thermometer and problem list scores at baseline (T0)

Distress thermometer	OncoAppstore users ***n*** (%) (***n*** = 347)
**Thermometer score** (mean (SD) median [IQR])^a^	5.6 (2.1)
	6.0 [4.0–7.0]
**Practical issues**	
Childcare	36 (10.4)
Housing	34 (9.8)
Housekeeping	173 (49.9)
Transportation	67 (19.3)
Work/school/study	178 (51.3)
Financial	48 (13.8)
Insurance	19 (5.5)
**Social issues**	
Dealing with partner	69 (19.9)
Dealing with children	43 (12.4)
Dealing with friends/family	100 (28.8)
**Emotional issues**^b^	
Keeping emotions under control	170 (49.1)
Memory	200 (57.8)
Self confidence	173 (50.0)
Fears	151 (43.6)
Depression	157 (45.4)
Tension/nervousness	202 (58.4)
Loneliness	106 (30.6)
Concentration	244 (70.5)
Feelings of guilt	118 (34.1)
Loss of control	152 (43.9)
**Religion/spiritual issues**^b^	
Meaning of life	56 (16.2)
Trust in God/religion	19 (5.5)
**Physical problems**	
Appearance	142 (41.0)
Changes in urination	39 (11.3)
Constipation	85 (24.6)
Diarrhoea	70 (20.2)
Eating	73 (21.1)
Feeling swollen	130 (37.6)
Fever	14 (4.1)
Mouth sores	44 (12.7)
Nausea	64 (18.5)
Nose dry/congested	97 (28.0)
Pain	171 (49.4)
Sexuality	136 (39.3)
Skin dry/itchy	113 (32.7)
Sleep	211 (61.0)
Shortness of breath/breathing	57 (16.5)
Dizziness	98 (28.3)
Speech/talking	30 (8.7)
Taste	54 (15.6)
Weight change	139 (40.2)
Tingling in hands/feet	148 (42.8)
Bathing/dressing	21 (6.1)
Daily activities	143 (41.3)
Fatigue	293 (84.7)
Out of shape/condition	263 (76.0)
Muscle strength	228 (65.9)
**Would you like to talk to someone about your problems?**^c^	
Yes	48 (14.0)
Maybe	118 (34.4)
No	177 (51.6)
**If yes, with whom?**^d^	
Nurse	44 (26.5)
Spiritual caregiver	13 (7.8)
Dietician	15 (9.0)
Psychologist	95 (57.2)
Fellow patients	37 (22.3)
Physical therapist	49 (29.5)
Social worker	23 (13.9)
Physician/oncologist	42 (25.3)
General practitioner	32 (19.3)
Assistant practitioner	22 (13.3)
Service centre for information and support	23 (13.9)
Other	28 (16.9)

OncoAppstore users: OncoAppstore users who completed baseline questionnaire (T0)

^a^The thermometer score is a visual analogue scale measuring general symptom burden, ranging from 0 (no burden at all) to 10 (extreme burden)

^b^1 missing value

^c^4 missing values

^d^Multiple options could be chosen; therefore, percentages do not add up to 100%

Abbreviations: *SD *standard deviation, *IQR* interquartile range

Furthermore, about half of the users reported that they (maybe) want to talk to someone about their problems (48.4%). Most users who reported this indicate that they want to talk to a psychologist (57.2%), physical therapist (29.5%), nurse (26.5%), physician/oncologist (25.3%), and/or fellow patients (22.3%).

### Employment status

The employment status of our study population is shown in Table 4. The employment status prior to the cancer diagnosis showed that most users were full-time employed (29.5%), part-time employed (40.4%), or self-employed (12.0%). After their cancer diagnosis, most users were either employed but on sick leave or retired (31.5% and 17.0%, respectively).

**Table 4:** Employment status

Employment status	OncoAppstore users ***n*** (%) (***n*** = 359)	
	Prior to diagnosis	After diagnosis
Full-time employment	106 (29.5)	39 (10.9)
Part-time employment	145 (40.4)	58 (16.2)
Self-employed	43 (12.0)	31 (8.6)
Unpaid work	11 (3.1)	11 (3.1)
Studying	13 (3.6)	6 (1.7)
Retired	44 (12.3)	61 (17.0)
Unemployed (involuntary)	5 (1.4)	4 (1.1)
Unemployed (voluntary)	13 (3.6)	16 (4.5)
(Partial) incapacity for work	13 (3.6)	43 (12.0)
Employed but on sick leave	4 (1.1)	113 (31.5)
Unemployed and on sick leave	0 (0.0)	17 (4.7)
Stay at home	10 (2.8)	12 (3.3)
Other	13 (3.6)	15 (4.2)

OncoAppstore users: OncoAppstore users who completed baseline questionnaire (T0)

Multiple options could be chosen; therefore, percentages do not add up to 100%

### EORTC CAT core

Table 5 shows the EORTC CAT Core scores. The European general population has a mean *T*-score of 50 (SD = 10). The users score more than one standard deviation lower than the general European population for role functioning (M = 38.1, SD (8.7), Md = 40.3, IQR [32.8–40.3]), cognitive functioning (M = 39.2, SD (9.2), Md = 41.8, IQR [32.8–41.8]), social functioning (M = 40.0, SD (8.1), Md = 42.0, IQR [34.7–42.0]), and the nausea/vomiting symptom domain (M = 78.0, SD (11.1), Md = 83.8, IQR [72.0–83.3]). Therefore, the means for these domains are below 84% of the scores from the general European population.

**Table 5:** EORTC CAT Core scores at baseline (T0)

EORTC CAT Core	OncoAppstore users (*n = 350)*	
	Mean (SD)	Median [IQR]
**Functioning scales**		
Physical functioning	43.3 (9.0)	43.5 [36.1–46.7]
Role functioning^a^	38.1 (8.7)	40.3 [32.8–40.3]
Emotional functioning^b^	46.8 (7.8)	45.0 [42.3–51.4]
Cognitive functioning^a^	39.2 (9.2)	41.8 [32.8–41.8]
Social functioning^a^	40.0 (8.1)	42.0 [34.7–42.0]
**Symptom scales**		
Fatigue^a^	59.5 (7.8)	61.1 [54.4–64.0]
Pain^a^	59.9 (8.3)	63.5 [55.5–63.5]
Sleep disturbances^a^	49.0 (6.3)	45.1 [45.1–54.8]
Nausea/vomiting^a^	78.0 (11.1)	83.8 [72.0–83.8]
Appetite^a^	52.8 (9.2)	46.8 [46.8–62.7]
Constipation^a^	51.4 (7.8)	46.3 [46.3–59.3]
Diarrhoea^a^	52.3 (9.4)	46.2 [46.2–62.0]
Dyspnoea^a^	50.8 (8.5)	45.2 [45.2–60.0]
Financial difficulties^a^	52.5 (8.3)	47.1 [47.1–61.0]

OncoAppstore users: OncoAppstore users who completed baseline questionnaire (T0)

Higher scores on the functioning scales indicate higher levels of functioning, whereas higher scores on the symptom scales represent greater symptom burden

^a^2 missing values

^b^3 missing values

Abbreviations: *SD* standard deviation, *IQR* interquartile range

**Fig. 2:**
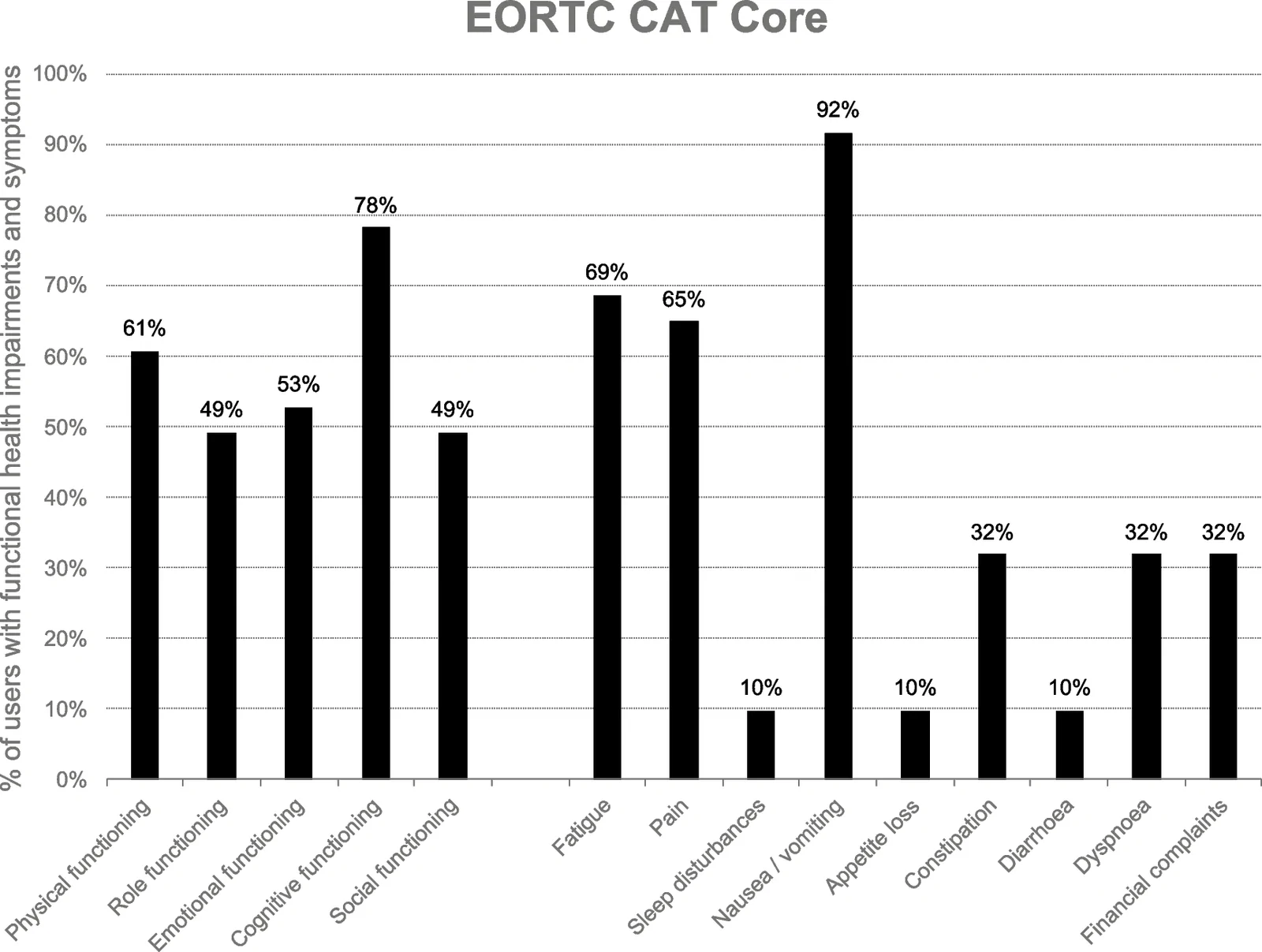
Percentage of users with clinically significant functional health impairments and symptoms based on thresholds for clinical importance [29] at baseline (T0, *n* = 350)

Furthermore, presented in Figure 2 are the percentages of users with clinically significant functional health impairments and symptoms. About half of the users score below the clinical threshold on all five functioning scales, indicating a high level of functional health impairments in the study population. This percentage is particularly high on the cognitive functioning scale, with 78% of users scoring below this threshold of clinical importance.

Contrary to the functioning scales, scores above the threshold on the symptom scales indicate clinically important symptoms (Figure 2). The symptom scales with the highest number of users scoring above the thresholds are the fatigue, pain, and nausea/vomiting symptom scales.

## Discussion

The OncoAppstore is, to our knowledge, the first platform to offer cancer-specific and cancer-relevant self-management applications for people directly affected by cancer and their relatives. In this case, it is specifically developed for the Dutch cancer population to improve access to these applications. This study, as part of the real-world OncoAppstore study, provides the first insights into the sociodemographic and HRQoL of the OncoAppstore user population.

In the first 15 months, 3620 users requested a health credit and purchased 4333 applications. On average, users utilised €53 of the available €100 health credit. Given that the implementation of healthcare interventions is estimated to take approximately 17 years, we are satisfied with the number of users reached in the first 15 months and expect a continued steady increase in user adoption going forward [30–33]. Users were predominantly highly educated females diagnosed with breast cancer who seek applications targeting cancer-related fatigue. Clinically significant distress was reported by the majority of users and approximately half indicated experiencing substantial functional impairments and expressed a need for support. Most users were employed before diagnosis, though many were on sick leave or retired afterward. Symptom burden was highest for fatigue, pain, and nausea/vomiting.

Previous research has highlighted disparities in healthcare utilisation, unmet needs, and health-seeking behaviours among people directly affected by cancer [34–40]. The use of healthcare services and/or health technologies is often associated with younger age, female gender, higher education level, employment, receiving chemotherapy, and higher levels of distress and depression [34–40]. Research suggest that age and distress are interrelated due to the responsibilities inherent to different life stages. Managing work and a cancer diagnosis can increase the need for additional support [37]. Similarly, gender and distress are interrelated. There are differences between men and women, both in levels of stress as well as coping strategies. Women experience higher levels of stress and they have a more emotional-focused coping style [41]. This aligns with our findings, as the majority of users in this study were distressed, females, and employed prior to their diagnosis.

Furthermore, our user population received more surgery, chemotherapy, radiotherapy, hormone therapy, and targeted therapy compared to the Dutch adult cancer population, which could be related to the differences in tumour site and tumour stage. These differences may have contributed to an increased symptom burden. More treatment could also have led to more contact moments with healthcare professionals and/or fellow patients. This could have enhanced the relationship with healthcare professionals and knowledge of additional care services, both of which are known facilitators to access to additional care services [42].

Additionally, one specific application, Untire Now, which focuses on cancer-related fatigue, was purchased by two-thirds of all users who purchased an application [8]. This is consistent with our HRQoL findings as most users reported experiencing problems with fatigue, concentration issues, and cognitive functioning. Decision aids to support treatment-related decisions and applications focused on the period from diagnosis to the end of treatment are well represented in the applications offered in the OncoAppstore, as well as applications targeting breast-cancer patients. These applications specifically attract users who were recently diagnosed and/or had breast cancer, which is reflected in our user population.

We consider it probable that individuals engaging with the OncoAppstore, and self-management applications in general, exhibit more proactive help-seeking behaviour. Their engagement is likely driven by unmet needs, significant functional impairments, and/or a high symptom burden.

Additionally, previous research indicates that people directly affected by cancer have limited awareness of self-management tools and/or additional care services [19, 43, 44]. This lack of awareness appears to be more prevalent in older individuals with a low socioeconomic position and lower education levels [43–45], a trend that is consistent with our findings. In recent years, psychosocial care has increasingly become an integral component of the cancer treatment. With nearly 30% of our users who became aware of the OncoAppstore through a healthcare professional, guidance from healthcare professionals to appropriate additional care services is an important facilitator of self-management adaptation in our study population as well. Further integrating psychosocial care and increasing awareness of additional care services is warranted.

### Study limitations

A strength of this study is the direct implementation of the OncoAppstore into a real-world setting, providing immediate access to self-management applications for all interested Dutch people directly affected by cancer and their relatives. The applications target some of the core issues that the majority of people directly affected by cancer are facing, such as cancer-related fatigue, pain, depressive symptoms, and improving lifestyle and knowledge [8–17]. Furthermore, to ensure quality, applications had to meet GGD assessment criteria, which include usability, reliability, privacy, and scientific foundation, before inclusion in the OncoAppstore [22]. The OncoAppstore operates within kanker.nl, a widely recognised online platform among people directly affected by cancer, their relatives, and healthcare professionals, attracting over one million visits per month and more than 39,500 users with an account. Unawareness of digital interventions and concerns over cost have been identified as barriers to self-management application uptake [19, 46]. By eliminating financial constraints and using a well-known platform, the OncoAppstore addresses these barriers, aiming to improve broader accessibility of online self-management tools. Insights from this study could further promote broader user inclusivity by specifically targeting the potential user not described in the user population of this study.

This study has several limitations. As part of an implementation project, the study period was predetermined rather than based on participant inclusion, and no sample size calculations were conducted. Additionally, participation in the study is voluntary for OncoAppstore users, resulting in sociodemographic, clinical, and questionnaire data being collected from only 10% of all users. This may introduce bias when characterising the user population and comparing users to non-users; e.g., it could be that specific groups of people directly affected by cancer, that are currently underrepresented in the OncoAppstore user population, do use the OncoAppstore but are less inclined to provide data. Moreover, the OncoAppstore appears to attract a distinct user population compared to the general Dutch cancer population. Efforts should be made to further enhance its visibility and extend its reach to engage more people directly affected by cancer who can benefit from self-management applications. However, the changing proportions of users who discovered the OncoAppstore through the platform kanker.nl versus healthcare professionals, friends and family, and other sources suggest that awareness of the OncoAppstore is already increasing. Additionally, one specific application focusing on cancer-related fatigue was purchased a lot more often than other applications [8]; thus, users of this application are overrepresented in this study. However, research shows that fatigue is one of the most common symptoms in people directly affected by cancer, especially in patients who receive chemotherapy and/or radiotherapy or patients with metastatic disease, where the prevalence of cancer-related fatigue (CRF) exceeds 75% [47]. Furthermore, data from the NCR of 2023 and 2024 concern preliminary data; hence, data from non-users is still subject to change. This study included solely completed registration from the NCR. The percentage of complete registrations from 2024 was 31%; for 2023, this was 81%, which is similar to the 84% from 2022 and 2021. This could explain some of the difference seen in time since diagnosis between users and non-users. Moreover, detailed data on application usage were not collected, limiting the ability to assess the effectiveness of specific applications or the OncoAppstore as a whole. As usage patterns vary between individuals, drawing definitive conclusions on impact remains challenging. Although, this allows users to personalise their selection and use of applications, reflecting real-world usage more accurately.

### Conclusion and practical implications

The OncoAppstore was created to enhance access to self-management tools for Dutch people directly affected by cancer and their relatives. During the first 15 months, users were primarily highly educated women diagnosed with breast cancer seeking applications targeting cancer-related fatigue. Findings from this study may offer valuable insights into the characteristics of users who are receptive to online self-management tools, contributing to the improvement of self-management infrastructure and its long-term integration into Dutch cancer care. Identifying characteristics of the users could help allocate resources to people directly affected by cancer who both need and seek this type of support, while recognising that others may require additional care and may be unwilling or unable to engage with digital tools. Notably, the OncoAppstore is not intended to replace care or reach all cancer patients and relatives in need of supportive care, but by providing self-management tools to those who are able to use it, we can potentially save scarce resources for those who are unable to profit from self-management alone. Further research is needed to evaluate the impact of the OncoAppstore on health-related quality of life and healthcare utilisation.

## Data Availability

The (aggregated) data collected during this study are available from the corresponding author upon reasonable request.
